# Long-term health care savings of preventing child maltreatment

**DOI:** 10.3389/fped.2025.1719755

**Published:** 2026-01-12

**Authors:** Vincent J. Palusci

**Affiliations:** Departments of Pediatrics and Forensic Medicine, NYU Grossman School of Medicine, New York, NY, United States

**Keywords:** Adverse Childhood Experiences, chronic conditions, cost benefit analysis, health care costs, prevention

## Abstract

**Introduction:**

While the harms child abuse and neglect have long been recognized, a growing body of knowledge has more recently developed about the longer-term harms during adulthood and the effectiveness of prevention programs. Research into the long-term effects of Adverse Childhood Experiences has provided a framework for calculating the potential cost savings for the health care system for reductions in adult chronic disease.

**Methods:**

This community case study analyzed health care costs of chronic disease conditions in adulthood in the U.S. State of Michigan to construct economic models of potential savings after reductions in total Adverse Childhood Experiences scores. National estimates, state-specific incidence data, and projections from the Adverse Childhood Experiences studies were used to calculate the population-based impact and specific costs and adult health care savings.

**Results:**

This analysis compared projected costs for evidence-based prevention programs (home visiting, newborn head trauma education, and pediatric primary care) and showed that a reduction of one Adverse Childhood Experience resulted in health care savings of more than $128 million annually ($55,816,424–$202,054,506) statewide for adult cancer, diabetes mellitus, heart disease, stroke, and pulmonary disease

**Discussion:**

Building on these analyses, this community case study concluded that the health care savings alone more than justify any costs associated with child maltreatment prevention in the health care system.

## 1 Introduction

### 1.1 Costs of maltreatment

While the harms child maltreatment (CM) have long been recognized, a growing body of knowledge has only more recently developed about the associated harms and costs in the health care system for children and adults. Decades of research show that investments in child abuse prevention yield substantial returns, reducing healthcare costs, criminal justice expenses, child welfare involvement, and lost productivity (1–4). Research into the effects of Adverse Childhood Experiences (ACEs) and other social determinants of health have provided a framework for building on these mixed and often fragmented models of health care to address the diagnosis, treatment and prevention of child abuse and neglect within the health system.

The financial costs of child abuse are staggering and are spread across systems. The Centers for Disease Control and Prevention (CDC) has estimated that the lifetime economic burden of new cases of child maltreatment in the United States in 2015 alone was approximately $428 billion (5). This figure places the costs of child abuse on par with other major public health crises such as heart disease and diabetes. Earlier analyses estimated that the average lifetime cost per victim was $210,012 (2). Furthermore, children who experience abuse have higher rates of injuries, chronic illnesses, and mental health conditions requiring treatment throughout their lives. Maltreated children often require special education services, contributing to higher per-pupil spending. Child welfare investigations, foster care placements, and support services generate significant expenses. Survivors of abuse frequently experience reduced educational attainment, unemployment, and diminished lifetime earnings, which lower tax revenues and economic growth. They have increased risk of involvement with the justice system, both as victims and offenders, leading to costs for policing, courts, and incarceration. Beyond these measurable costs, there are intangible losses—pain, suffering, and reduced quality of life—that economists cannot easily quantify but are nonetheless profound.

### 1.2 Adverse childhood experiences

Adverse Childhood Experiences (ACEs) are events occurring before age 18 years that have lasting effects on health and well-being (6). The concept emerged from the landmark CDC–Kaiser Permanente ACE Study in the late 1990s, which surveyed over 17,000 adults about their childhood experiences and current health (7–9). The researchers identified 10 key categories of adversity grouped into three broad domains: abuse, neglect, and household dysfunction (Table 1). These experiences include direct maltreatment, such as physical, sexual, or emotional abuse, and neglect, as well as indirect stressors like growing up with substance abuse, mental illness, incarceration, or domestic violence in the home. Adverse Childhood Experiences (ACEs)—including abuse, neglect, and household dysfunction—are now recognized as major public health drivers of disease, disability, and social disadvantage (10). ACEs are common; national surveys suggest that nearly two-thirds of adults report at least one ACE, and about one in six report four or more. ACEs impose enormous economic costs on health care systems, social services, education, and the criminal justice system (3, 11, 12).

**Table 1:** Adverse Childhood Experiences (ACEs).

Domain	Specific ACEs
Abuse	Physical abuse; Emotional abuse; Sexual abuse
Neglect	Physical neglect; Emotional neglect
Household Dysfunction	Parental separation/divorce; Household substance abuse; Household mental illness; Incarcerated household member; Witnessing domestic violence

The link between early childhood experiences and lifelong outcomes is well established (1). Abuse and neglect undermine children's capacity to learn, form relationships, and contribute to society. For a single condition, Ortiz et al. (13) calculated that greater child abuse and decreased caregiver warmth were associated with 12.8% higher rates of cardiovascular illness. Nobel laureate James Heckman's work on early childhood interventions underscores that preventing adverse experiences yields higher returns than remediation later in life (14). The economic burden of ACEs has been estimated in the hundreds of billions annually, and economists highlight that early investments in children produce some of the highest returns of any social spending (4). Peterson et al. calculated that in 2019 and 2020 an estimated 160 million of the total 255 million U.S. adult population (63%) had 1 or more ACE, associated with an annual economic burden of $14.1 trillion ($183 billion in direct medical spending and $13.9 trillion in lost healthy life-years). This was $88 000 per affected adult annually and $2.4 million over their lifetimes. Twenty-two percent of adults had 4 or more ACEs and comprised 58% of the total economic burden—the estimated per person lifetime economic burden for those adults was $4.0 million. A global analysis across North America, Europe, and Asia found that ACEs accounted for an estimated 1.33% of gross domestic product in North America, largely due to health care costs and productivity losses. Reducing ACEs therefore has the potential to generate significant cost savings at both state and national levels. In the United States alone, annual ACE-attributable costs were estimated at more than $748 billion in 2017 (15).

Health-related costs arise because exposure to multiple ACEs is associated with an increased likelihood of costly outcomes, such as chronic disease, substance use disorders, mental illness, and premature mortality. Preventing ACEs offers opportunities for cost savings across several sectors, but health care is the most direct area of savings since individuals with high ACE scores have higher rates of hospitalizations, emergency department visits, and chronic disease management needs. Most adults in the U.S. (60%) have one or more chronic diseases and these are increasing in incidence (16, 17). Reducing ACE exposure would lower demand for expensive acute care and long-term treatment, particularly for cardiovascular disease, diabetes, and depression, all of which are strongly associated with early adversity. Additionally, the opioid epidemic has highlighted the link between ACEs and substance misuse, and prevention could save billions in addiction treatment and reduce overdose deaths.

### 1.3 Benefits from prevention

A number of analyses consistently show that while prevention programs require upfront funding, they pay off substantially in avoided costs. Estimates suggest that every dollar invested in early childhood interventions—such as home visiting and comprehensive pediatric primary care—returns multiple dollars in reduced costs and increased productivity (18–20). Moreover, the benefits extend beyond direct fiscal savings to include improved educational achievement, workforce participation, and health outcomes. Rigorous economic evaluations reinforce the financial rationale for prevention, and preventing and mitigating ACEs requires a comprehensive public health approach. Primary prevention strategies focus on strengthening economic supports for families, promoting positive parenting, and ensuring safe, stable, nurturing relationships and environments. Secondary and tertiary strategies emphasize trauma-informed care in health, education, and social service systems, early intervention for children exposed to adversity, and community-based programs to foster resilience. Current health care initiatives, such as integrating ACE screening in pediatric and family medicine, are being tested to balance early identification with the need for supportive, non-stigmatizing responses.

Policy initiatives such as expanding home visiting, increasing family economic supports, and integrating trauma-informed care into schools and health systems have demonstrated effectiveness in reducing ACE exposure (21). Peterson et al. (5) assessed the U.S. state-level budget and societal impact of implementing two child abuse and neglect primary prevention programs (Child-Parent Centers and Nurse-Family Partnership) to compare program costs with the future monetary value of saved health care costs and increased tax revenues from reduced child maltreatment, finding savings using a lifetime time horizon from government payer and societal perspectives. Other programs such as pediatric primary care and new parent education on responding to crying also offer potential reductions for certain forms of maltreatment. The costs for these programs pale in comparison to health care costs, which years later will rise substantially more than the consumer price index. Health care costs rose 50% during 2010 through 2025 while the consumer price index rose 43% over the same time period (22, 23).

### 1.4 Prevention strategies

Several health-related strategies have been shown to produce both improved outcomes and economic savings (24):

1. Home Visiting Programs: One of the most studied interventions is nurse home visiting for at-risk families. The Nurse-Family Partnership (NFP) in which nurses visit low-income first-time mothers during pregnancy and early childhood, has demonstrated long-term reductions in child maltreatment. One review of 15 early childhood intervention studies (birth to age 5y) found a weighted average effect size equivalent to a 42% reduction in the rate of maltreatment cases (24). Economic evaluations show that NFP produces a return on investment of up to $5.70 for every $1 spent, largely through reduced child welfare involvement, healthcare costs, and increased maternal employment (18, 20). Karoly et al. (18) at the RAND Corporation found that NFP generated net benefits of $17,000 per child served when implemented with high-risk populations even after estimated per family costs of $2,000 or more. These benefits accumulated from reduced welfare dependency, improved child outcomes, and lower criminal justice involvement. In Michigan in 2002, Noor and Caldwell (25) estimated that a prevention program where every Michigan family having their first child either received a comprehensive parent education program or a home visitor would cost approximately $48.87 million, or 2.7% of the cost of CM.
2. Parent education on newborn crying and the dangers of shaking: The Period of PURPLE Crying program aims to prevent abusive head trauma by educating caregivers about crying and has been linked to a 30% reduction in physical abuse hospitalizations for children under two years in British Columbia, Canada (26). Cost estimates from our implementation of a similar program were estimated at approximately $20 per family with a 25% reduction in later abusive head trauma (27)
3. Pediatric Primary Care: The Safe Environment for Every Kid (SEEK) program is a model to identify and help address prevalent psychosocial problems that are risk factors for child maltreatment. The primary targets include parental depression, substance use, stress, intimate partner violence, harsh punishment and food insecurity. SEEK was initially implemented and tested in an urban pediatric resident clinic serving a high-risk population; 33% fewer families in the intervention group were reported to CPS compared to control families who received routine care (19, 28). It has also been tested in private practice primary care. Costing approximately $4 per child in addition to routine pediatric care and based on a very conservative cost estimate of $2,779 per maltreatment incident, SEEK would have saved an estimated $2,151,878 in health care costs for 29,610 children (19).

### 1.5 Calculating program costs and health savings

Using population attributable risk fraction (PARf) calculations, changes in the numbers of persons with specific chronic conditions can be calculated based on information from prior ACEs studies (8, 9, 29). These studies provide different risk ratios based on total ACE score, with a total score of four being associated with the greatest increase in the disease incidence. Annual estimates of costs associated with those conditions in the U.S. can then be summed and adjusted based on a decrease of total ACE score, such as from four to three (22). This can be adjusted based on the known effectiveness of specific prevention programs in reducing one or more forms of child maltreatment (and therefore ACE scores) available from program reports. The decreases in those costs can then be compared with the annual costs associated with providing child maltreatment prevention programming to appropriate segments of the entire population. Subtracting those costs from the chronic disease savings would yield an estimate of at least some of the potential annual economic benefits of prevention in the health care sector. This can be calculated using the following Equations 1–5:

Riskratioforachronicmedicalcondition(RR4v3)=RR(4v0)/RR(3v0) (1)

Populationattributableriskfraction(PARf4v3)=((RR(4v3)–1)/RR(4v3) (2)

Medicalsavings(MS)=Totalconditioncost×PARf(4v3) (3)

$$\begin{array}{rl} & \;\mathrm{Adjusted}\;\mathrm{medical}\;\mathrm{savings}\;(\mathrm{aMS})\\ & =\mathrm{MS}\times \mathrm{intervention}\;\mathrm{effectiveness}\;(\text{\%}\mathrm{CM}\;\mathrm{reduction}) \end{array}$$ (4)

Netannualsavings=aMS–annualcostsofpreventionprograms (5)

## 2 Setting and population

The U.S. state of Michigan was chosen for this study based on its large, mixed population, high prevalence of chronic disease, and accessible health and census data. Michigan overall has had chronic condition prevalence estimates which were similar to the U.S. as a whole (30). The year 2010 was chosen for its most complete health and census data without effects from COVID-19. In 2010, Michigan's total population according to the U.S. Census had approximately 2,056,000 children under 18, representing a significant portion of the state's demographic base with implications for service needs in areas such as education, health, and child welfare (31). Rural vs. urban differences shaped children's lives; many children in urban centers had different access to resources than those in rural counties. There were 113,087 live births and 539,247 families with children (32). While the raw number of births had been declining somewhat compared to earlier years, the health conditions surrounding birth remained inequitable; mothers from disadvantaged populations were more likely to have risk factors such as inadequate prenatal care, higher prevalence of health conditions (e.g., hypertension, diabetes), and greater exposure to stressors associated with poverty, which contribute to adverse birth outcomes (33). Estimates of economic costs for health care are in excess of $10 billion annually and are broken down in Table 2 (22).

**Table 2:** Michigan health care costs.

Metric	2010 (or nearest)	2025 (or most recent)	Notes/Data Source
Michigan per capita health expenditure (personal health care)	$6,618 in 2009	–	The 2009 figure from Michigan's state health expenditure report is a close proxy for 2010. (33)
Michigan per capita health care consumption	–	$8,381 (2023)	2023 is the latest available in for Michigan. (34)
Michigan Medicaid spending change per enrollee	–	+18% growth in spending per enrollee since 2003.	This provides a growth rate, not an absolute dollar amount. (35)
Michigan hospital funding/uncompensated care	–	Michigan hospitals receive nearly $7 billion in Medicaid funding annually.	This is a current scale volume metric, not strictly comparable to 2010. (36)

This study involved secondary analysis of publicly available, de-identified documents and did not involve human subjects. Ethical review was not sought as all of the information used was available in the public domain.

## 3 Key programmatic elements

Using the Equations 1–5 and data from Michigan for 2010, we can calculate the annual costs for prevalent adult chronic conditions (Cancer, Diabetes Mellitus, Heart Disease, Stroke, and Pulmonary Disease) and compare them with the costs of three prevention programs (home visiting using NFP, new parent education using the Period of Purple Crying program, and pediatric primary care using the SEEK model). These chronic conditions were chosen because of their prevalence, high costs and inclusion in published ACEs studies. The prevention programs were included because of their current use in health care systems and available published characteristics.

The calculations for Michigan in 2010 were as follows (Table 3). The risk ratio (RR) for a chronic medical condition with four ACEs was compared to the risk with three ACEs in Equation 1. The population attributable risk fraction (PARf) then determined the proportion of the Michigan population affected by the condition of interest, allowing the number of individuals to be calculated based on the decreased proportion (Equation 2).

**Table 3:** Population attributable risk fraction for medical conditions.

Disease	RR (4 v 0)	RR (3 v 0)	RR (4 v 3)	PARf (4 v 3)
CA	1.9	1.0	1.900	0.017
DM	1.6	1.2	1.333	0.012
HD	2.2	1.4	1.571	0.027
PD	4.6	2.6	1.769	0.091
ST	2.4	1.3	1.846	0.004

Equation 1. Risk ratio for a chronic medical condition; Equation 2. Population attributable risk fraction.

CA, cancer; DM, diabetes mellitus; HD, heart disease; ST, stroke; PD, pulmonary disease.

Medical savings (MS) for a particular condition is calculated based on the change in the ACE score from four to three which decreases the population affected by a condition, resulting in decreased annual health care costs. This total condition cost is calculated from the condition cost in the U.S and the condition prevalence in the U.S. and Michigan. MS in Michigan for 2010 was calculated to be $1,462,380,821 (Equation 3) (Table 4). This was adjusted using an assumed 25% effectiveness rate with a range of 20%–30% based on previous studies, revising the aMS to $365,595,205 ($292,476,164–$438,714,246) (Equation 4). Costs for prevention programs totaled $236,659,740 based on published estimates for typical programs, and multiplied by the annual number children it would be provided for (113,087 infants and newborns and 2,056,000 children) (32) (Table 5). These costs, when deducted from projected health costs savings, yielded the net annual savings of $128,935,465 annually ($55,816,424–$202,054,506) after subtracting the costs of the prevention interventions (Equation 5).

**Table 4:** Medical savings based on change in ACEs score and prevention effectiveness.

Disease	DisPrev (US)	DisCost (US, 2010)	DisPrev (state)^a^	Total condition	PARf (4 v 3)	Cost (4 v 3)
CA	0.037	$ 73,230,000,000	0.036	$2,636,280,000	0.017052632	$44,955,512
DM	0.049	$ 41,380,000,000	0.046	$1,903,480,000	0.0115	$21,890,020
HD	0.068	$ 95,680,000,000	0.074	$7,080,320,000	0.026909091	$190,524,975
PD	0.174	$ 62,810,000,000	0.21	$13,190,100,000	0.091304348	$1,204,313,478
ST	0.009	$ 18,770,000,000	0.009	$168,930,000	0.004125	$696,836
				$24,979,110,000	MS=	$1,462,380,821
						aMS
				Adjusted MS	20%	$292,476,164
					25%	$365,595,205
					30%	$438,714,246

CA, cancer; DM, diabetes mellitus; HD, heart disease; ST, stroke; PD, pulmonary disease.

a Disease Prevalence, Michigan State data (http://www.chronicdiseaseimpact.com/).

**Table 5:** Costs for prevention programs.

Strategy	Unit cost	Target	Target population	Program cost
NB Education	$20	Newborns	113,087	$2,261,740
Primary Care	$4	Children	2,056,000	$8,224,000
Homevisiting	$2,000	Infants	113,087	$226,174,000
				$236,659,740

## 4 Discussion

This analysis calculated the decreased annual costs for a limited number of adult chronic health conditions and subtracted the costs of three health-related prevention programs, yielding a savings of $55–202 million, roughly $6–20 for each of the approximately 10 million Michigan residents in 2010. In a prior comprehensive savings analysis, Robert Caldwell compared the costs associated with child maltreatment and its consequences with the cost of providing child maltreatment prevention services in a 1992 state-level analysis (37). The costs of child abuse in Michigan were estimated at $823 million annually, and the costs of prevention programming varied depending on the intensity of the services offered but costs were significantly less than the costs of treating the consequences. In a 2002 reanalysis (25), the costs of child abuse in Michigan were estimated at $1,827,694,855, but costs of prevention programming were still just a fraction of the child abuse treatment costs. Cost savings ranged from 96% to 98% depending on the prevention model tested; however, long term medical costs were not included in their calculations. Beyond health care, another study showed that the economic burden of known child maltreatment cases in 2018 in Michigan was $32,142,622,498, with 158,673 investigated cases, 37,703 victims and 49 child deaths (38).

The economic case for child abuse prevention has clear policy implications. Policymakers should allocate stable funding streams for evidence-based prevention programs, recognizing them as cost-saving public investments. This means that prevention should be integrated into healthcare, education, and social services to maximize reach and efficiency and to distribute costs and savings, including expanding economic supports for families as both an anti-poverty and a child abuse prevention strategy. Evaluations should account for benefits across the life course rather than focusing narrowly on immediate outcomes. If these later savings could be captured within the health care system, health care CM prevention activities could be justified.

In calculating cost savings, one should also keep in mind the 15–40 year time lapse between ACE exposure during childhood and the appearance of chronic conditions in adults, the median age for which is age 45 years (16). Health costs will rise over that time lapse. Michigan's per capita health expenditures in 2009 were about $6,618 (33). In 2025, state budget documents and related health-policy data suggest that Michigan's health spending burdens had grown substantially to $9,900 per person. The National Health Spending Explorer shows growth across the decades, with per capita national health spending rising at an average annual rate of ∼5.4% through 2028 (39, 40). Michigan Medicaid spending per enrollee has increased, though more slowly (18%) compared to national health insurance premium growth, signaling that Michigan's health cost pressures are real but moderated in that domain (33). In the private health insurance space, one estimate suggests an 8% year-on-year medical cost trend in 2025 for the group market, and 7.5% for the individual market—indicating steep cost escalation (41). Hospital costs in Michigan have risen dramatically; one commentary states that between 2010 and 2020, payers in Michigan experienced a 40% increase in costs for hospital services (36, 42). Taken together, these suggest that by 2025, Michigan's per capita health spending is likely well above $9,000 reflecting inflation, utilization growth, medical wage rises, and new technologies. These and other cost projections can only increase the savings based on prevention given that health care costs generally rise faster than the consumer price index (43).

## 5 Limitations

Despite robust available evidence, several challenges complicate the economic analysis of child abuse prevention. This study has made a number of assumptions based on national and state data which, while conservative, may have overestimated savings from prevention. Foremost, the analysis assumes that the total ACE score can be modified by intervention and that the association of ACEs with later health is robust and not dependent mostly on confounders such as poverty, which likely account for some of the effects. ACEs can plausibly capture more difficult to observe forms of maltreatment that reflect household dysfunction, such as supervisory and emotional neglect, that likely also bias the results. It also assumed that prevention programs are at least 25% effective in reducing the ACEs score of the average Michigan adult by one point, specifically from four to three. A reduction in the ACEs score of this nature may not yield equivalent effects of preventing child abuse and neglect, thereby reducing the effectiveness of intervention. Furthermore, the use of a single, average estimated effect for each prevention program fails to capture variation in program effects across demographic characteristics, such as SES, parental age, and race/ethnicity; for high-risk populations, the estimated reductions are likely larger in magnitude.

Program effectiveness depends on fidelity to evidence-based models, and costs can vary across settings, which will vary state to state and even within the state. Not all studies of the Period of Purple Crying, for example, have yielded positive prevention results (44). Adults may come to the state who have not been provided these programs as children and who will therefore have increased ACE scores and more chronic disease. Alternatively, challenges in measurement and limiting the cost savings to medical costs associated with five chronic health conditions may mean that these estimates may actually understate the true economic value of prevention since many benefits, such as reduced suffering or improved family relationships, are difficult to monetize. An analysis of the combined effects on health care costs of multiple chronic diseases is also beyond the scope of this report, as individuals with more than one of the five measured diseases may have not have additive health care costs, reducing projected savings. This analysis also cannot account for the myriad benefits of ACEs reduction which were not measured for additional chronic health conditions as well as other physical and mental health issues which will have significant costs in the health system later in life. Benefits will accrue decades after prevention efforts, while funding cycles operate on short-term horizons, and economic analyses may not fully capture the societal value of reducing disparities in maltreatment risk over time, modern income inequality rates, the shift to a work-based safety net, and rising costs of childrearing that are unique to modern parents. Similarly, program costs might have fallen due to increased efficiency and improved monitoring. They are also subject to variations based on local and state factors and the economic vagaries of health care economics (45). However, given standard practices for financial commitments of 30 years or more for bonds and mortgages, one can only hope there is at least an equivalent value and justification in our society for investing in our human capital.

## 6 Conclusion

Child abuse imposes profound costs on individuals and society. Yet these costs are not inevitable. Evidence-based prevention programs demonstrate that targeted investments yield substantial returns, saving money while improving the lives of children and families. This shows what we have long known: preventing maltreatment is not only the right thing to do but also the fiscally responsible choice. By investing in prevention, society can reduce the multibillion-dollar burden of child maltreatment, promote healthier families, and secure a stronger economic future. ACEs represent both a profound challenge and a powerful opportunity to accrue future savings in the health care system (46). While implementation to reduce them requires upfront investment, the return in avoided health expenditures, reduces social costs, and enhanced productivity makes ACE prevention one of the most cost-effective strategies in private and public health. Preventing abuse before it occurs avoids downstream expenditures and allows children to thrive. This analysis of Michigan data shows that reducing childhood adversity could save the United States billions of dollars annually in health care costs alone for a limited number of chronic conditions in adulthood. Future research can expand on the health conditions considered, the prevention programs implemented, the reductions in chronic conditions associated with other reductions in ACEs, and the more specific cost savings achieved in communities in the state in more recent years. This limited analysis shows that preventing child abuse and neglect reduces future long term health care expenditures in addition to paying dividends that extend across lifetimes.

## Data Availability

The original contributions presented in the study are included in the article/Supplementary Material; further inquiries can be directed to the corresponding author/s.
